# Pediatric Chondrosarcoma of the Sternum Resected with Thorascopic Assistance

**DOI:** 10.2174/1874325001711010479

**Published:** 2017-05-31

**Authors:** Harpreet S. Bawa, Drew D. Moore, Juan C. Pelayo, Nicole Cipriani, Grace Mak, Rex C. Haydon

**Affiliations:** 1 Department of Orthopaedic Surgery and Rehabilitation Medicine University of Chicago Division of Biological Sciences 5841 South Maryland Avenue MC 3079 Chicago, IL 60637, USA; 2 Department of Orthopaedic Surgery William Beaumont Hospital 3535 West 13 Mile Road, Suite 704 Royal Oak, MI 48073, USA; 3 Children's Hospital Los Angeles Division of Pediatric Surgery 4650 Sunset Blvd., #100 Los Angeles, CA 90027, USA; 4 Department of Pathology University of Chicago Division of Biological Sciences 5841 South Maryland Avenue MC 4062 Chicago, IL 60637, USA; 5 Department of Pediatric Surgery University of Chicago Division of Biological Sciences 5841 South Maryland Avenue MC 4062 Chicago, IL 60637, USA

**Keywords:** Chondrosarcoma, Chest wall mass, Video-assisted thoracoscopic surgery, Sternal tumor, Sarcoma

## Abstract

**Background:**

Chondrosarcomas are a heterogeneous group of malignant neoplasms that arise from bones, cartilage or other soft tissues that produce cartilage and are commonly seen in the middle decades of life. Despite being the most common primary bone sarcoma in adults, chondrosacromas are rare in pediatric patients.

**Case Report:**

We report the case of a six-year-old child with a painless enlarging sternal mass of which biopsy was consistent with low-grade surface chondrosarcoma. This is the first reported case of a chest wall chondrosarcoma in a young child. This unusual location in a young patient presented challenges to treatment. Resection of the manubrium was performed by a multidisciplinary team of orthopaedic oncology and pediatric general surgery. The patient underwent a wide resection of the sternal mass from an anterior approach performed by the orthopaedic oncology team using an oscillating saw under video-assisted thoracoscopic surgery to ensure adequate mass resection without injury to nearby structures. The patient was followed with quarterly physical exams and radiographs for 18 months postoperatively and did not have any pain or evidence of recurrence.

**Conclusion:**

Clinicians should consider utilizing multidisciplinary approaches to treat patients with chondrosarcomas of the chest wall.

## INTRODUCTION

Chondrosarcomas are a heterogeneous group of malignant neoplasms that arise from bones, cartilage or other soft tissues that produce cartilage and are commonly seen in the middle decades of life [1, 2].

They most commonly involve flat bones of the pelvic and shoulder girdle, however approximately 15% present in the chest wall with annual incidence of 0.5 per million [3].

They are rarely seen in young patients and to our knowledge no reports exist of a chondrosarcoma of the chest wall in a young child. A series of 89 patients with primary chest wall chondrosarcomas had a median age of 55 years and with the youngest being 15 years of age [4].

Chondrosarcomas provide unique challenges to treatment. While intralesional curettage has been shown to be effective for low grade lesions; intermediate or high grade tumors typically require wide excision [5-7]. This poses unique concerns for chest wall tumors as chest wall lesions greater than five centimeters likely require chest wall reconstruction [8]. Total sternal resection should be avoided to prevent chest wall instability and respiratory complications [9].

We present a unique case of a six-year-old with chondrosarcoma of the sternum successfully treated with wide resection with video-assisted thoracoscopic surgery (VATS).

The patient and family provided informed consent for the publication of this case report.

### CASE REPORT

**Fig. (1):**
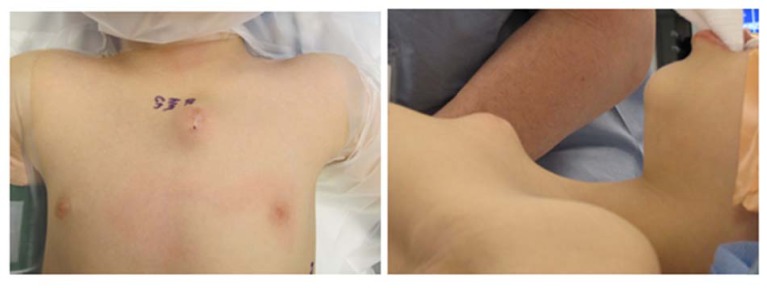
Pre-operative images of the patient’s sternal mass.

A six-year-old male was referred to the orthopaedic oncology clinic after initially presenting to a general surgeon regarding a mass on the left aspect of the manubrium. According to the patient’s parents, the mass was present since the child was two years old. It started as a pea-sized prominence and enlarged gradually. The patient denied any associated pain. There was no known history of trauma or injury. He denied any fevers, chills, sweats, or shortness of breath. At presentation, there was a three-centimeter palpable mass overlying the left side of the manubrium. This area was non-mobile and non-tender to palpation (Fig. **1**). He was neurovascularly intact in bilateral upper extremities. The patient was otherwise healthy and the remainder of physical exam including cardiopulmonary auscultation was unremarkable.

**Fig. (2):**
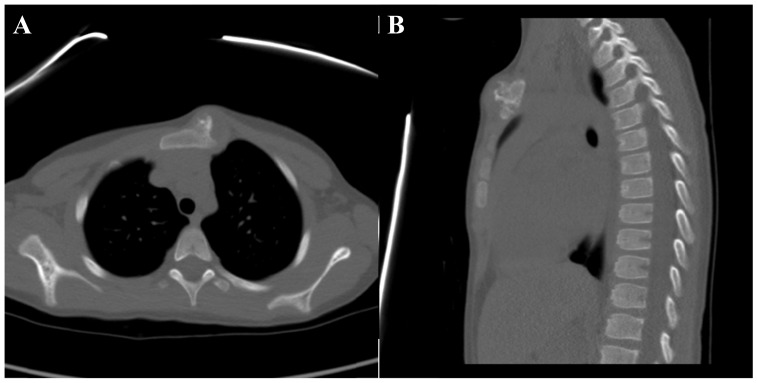
CT scan of the chest at initial presentation, including axial Fig. (**2A**) and a sagittal Fig. (**2B**) views.

Plain radiographs demonstrated a mass that extended into the soft tissues anterior to the manubrium with partially ossified edges. A computed tomography (CT) scan showed a bony exostosis arising from the left anterior aspect of the manubrium with a cartilage cap and internal mineralization. No underlying marrow changes were noted (Fig. **2**). Based on the patient’s history, physical exam, and imaging studies the differential diagnosis included osteochondroma, surface chondroma, or periosteal chondrosarcoma. On serial monitoring radiographs and a repeat CT scan eight months after presentation, the lesion was found to be gradually enlarging. At that time, after a discussion with the patient’s family a decision was made to perform an open biopsy of the lesion through a direct anterior approach.

**Fig. (3):**
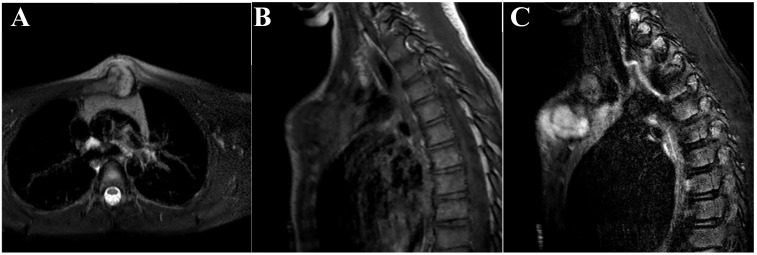
MRI scan of the chest after biopsy. (A) T2 weighted axial slice, (B) T1 weighted sagittal slice, and (C) short tau inversion recovery (STIR) sagittal slice of the chest.

The patient underwent open biopsy of the sternal mass under general anesthesia. It was performed *via* a small longitudinal incision directly over the anterior soft tissue mass to allow subsequent biopsy tract excision if needed. The biopsy revealed a cartilaginous neoplasm with variably hypo-cellular to moderately cellular areas and zonal necrosis. There was a portion of patchy chondrocyte atypia, including nuclear enlargement and hyperchromasia. No mitoses were identified. Spindled histocytes within the lesion were found to be positive for CD68 and lesional cartilaginous cells positive for S100. These findings favored a diagnosis of a low-grade surface chondrosarcoma. Following the biopsy magnetic resonance imaging (MRI) demonstrated a lesion with heterogeneous internal enhancement without an associated vascular component. The mass was seen extending posteriorly with abutment of the left thymus and great vessels (Fig. **3**). Given the histology as well as the aggressive features on MRI there was concern for an intermediate grade lesion, therefore a complete resection was recommended. Pediatric general surgery was consulted given the location of the lesion to ensure the deep structures posterior to the sternum were not compromised during resection.

Imaging demonstrated the mass was adjacent to the great vessels and internal mammary artery therefore safe resection required dissection of soft tissues prior to osteotomy. Wide resection of the lesion was performed under general anesthesia. Pediatric general surgery performed a left thoracoscopic dissection and mobilized the posterior portion of the mass away from the internal mammary artery and thymus. Next, the patient underwent a wide resection of the sternal mass from an anterior approach performed by the orthopaedic oncology team using an oscillating saw under direct thoracoscopic visualization; this ensured adequate mass resection without injury to nearby structures. Fig. (**4**) shows the chest wall after resection and Fig. (**5**) shows the resected specimen. The location and size of the defect did not require reconstruction. A chest tube was placed by the pediatric surgery team and patient was admitted for monitoring. His postoperative course was uncomplicated. The chest tube was removed on post-operative day two and he was discharged home in stable condition on post-operative day three.

**Fig. (6):**
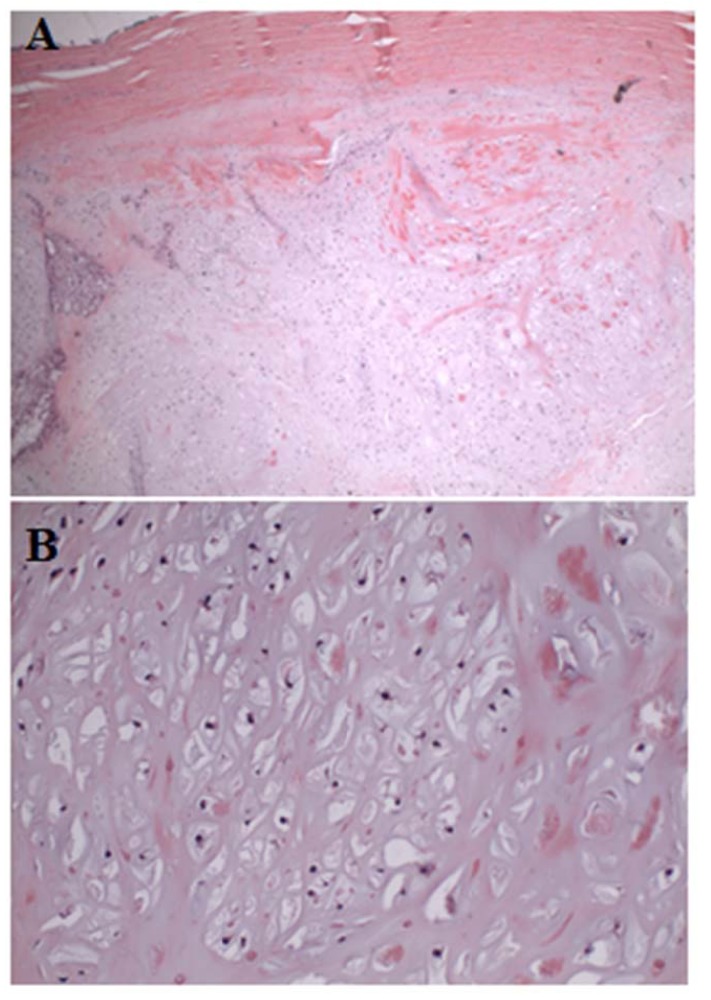
(A) Low power view of low cellularity cartilaginous neoplasm pushing into marrow space (left) and circumferentially entrapping bone (right). (B) A high-power view of chondrosarcoma circumferentially entrapping pre-existing lamellar bone, which has also undergone osteonecrosis.(C) Chondrocyte hypercellularity and cytologic atypia in the setting of bony entrapment.

**Fig. (7):**
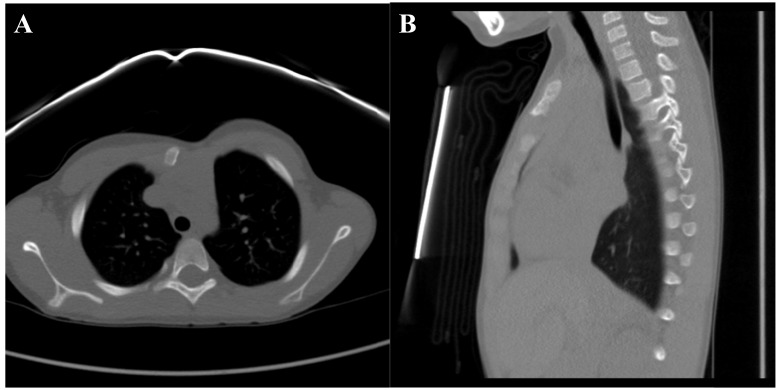
Follow up CT scan of the chest including axial Fig. (**7A**) and a sagittal Fig. (**7B**) views.

Final pathology demonstrated neoplastic cartilage surrounding pre-existing trabecular bone with associated osteonecrosis consistent with low grade chondrosarcoma with microscopically negative margins and closest being at 2 millimeters from the superficial skin (Fig. **6**). Given the complete excision, no adjuvant treatment was recommended. The patient was followed with quarterly physical exams and radiographs and as of 18 months postoperatively has no pain or recurrence noted on a most recent chest CT scan (Fig. **7**). Per National Comprehensive Cancer Network guidelines, the frequency of surveillance will decrease with time, but should be 10 years at minimum [10].

## DISCUSSION

This case report presents a six-year-old patient with a painless low grade chondrosarcoma of the sternum that progressively enlarged over four years. Chondrosarcomas are typically seen in adults. Although previous studies have evaluated primary chondrosarcomas of the chest wall, none have included patients under the age of 15 [4, 11]. Furthermore, this unusual location in a young patient presented challenges to treatment. A novel multi-disciplinary approach was utilized to perform a wide resection of the lesion.

The pediatric general surgery team performed a thoracoscopic mobilization of the posterior aspect of the tumor combined with resection of the manubrium. This unique approach allowed excellent visualization of both the anterior and posterior portions of the tumor ensuring adequate resection without injury to surrounding structures. Given that only four centimeters of the manubrium was removed and the sternum remained intact, a reconstruction was not necessary. The patient has done well post-operatively with no functional limitations or pain. Previous published reports have used five centimeters as the maximum amount of chest wall that can be resected without reconstruction [2, 6, 8, 9, 12].

Chondrosarcomas of the chest wall are rare and few studies have been performed evaluating optimal treatment. These lesions are often treated by general or thoracic surgeons who are not in larger sarcoma centers, which may lead to variability in management. Due to the challenges of establishing the correct diagnosis, it is important to utilize a multidisciplinary approach for treatment of each patient with a team of experienced radiologists, pathologists, medical oncologists and orthopaedic oncologists. A Scandinavian study evaluated 106 chest wall chondrosarcomas and found that approximately 60% were treated at general hospitals as opposed to sarcoma centers. Patients treated at non-sarcoma centers had higher rates of recurrence and lower survival [13].

## CONCLUSION

This case report presents a painless chondrosarcoma in a young patient. The VATS allowed lesion resection with minimal morbidity and with adequate margins. Clinicians should consider utilizing multidisciplinary approaches to treating patients with chondrosarcomas of the chest wall.

## Figures and Tables

**Fig. (4):**
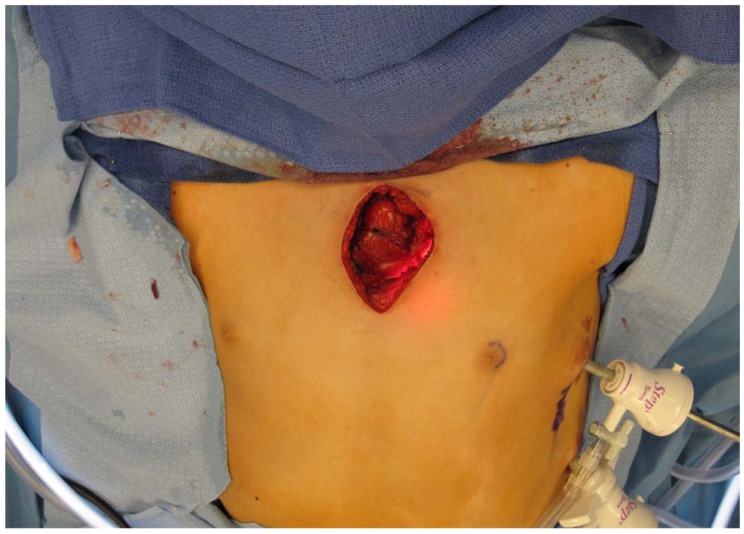
Intra-operative image of the chest wall after resection.

**Fig. (5):**
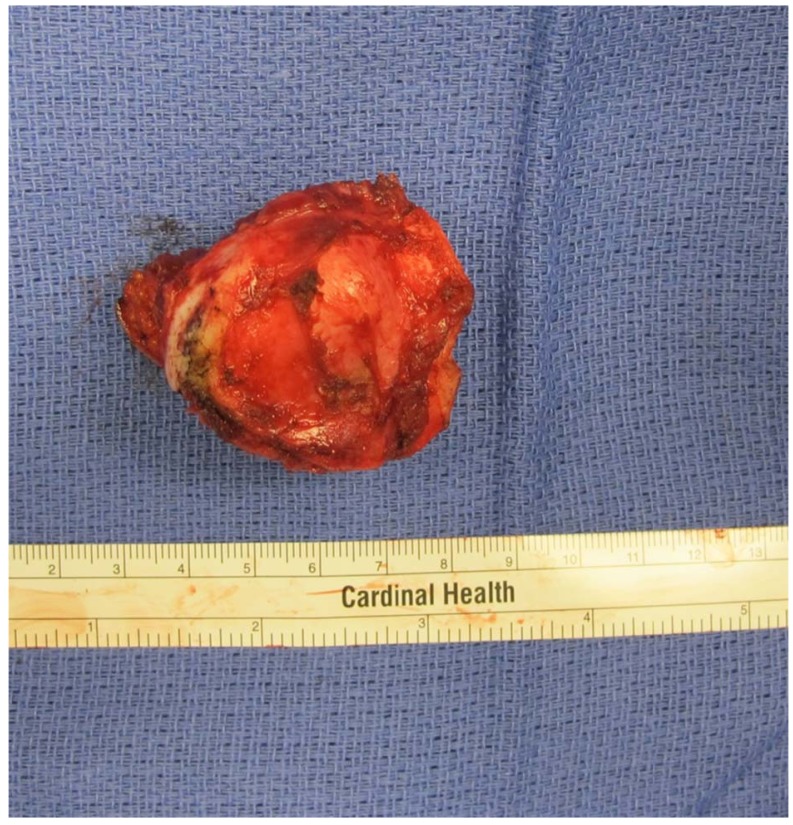
Image of the gross resected specimen.

## LIST OF ABBREVIATIONS

CT: = Computed tomography
MRI: = Magnetic resonance imaging
STIR: = Short tau inversion recovery
VATS: = Video-assisted thoracoscopic surgery
